# Mechanisms of Antimicrobial Action of Cinnamon and Oregano Oils, Cinnamaldehyde, Carvacrol, 2,5-Dihydroxybenzaldehyde, and 2-Hydroxy-5-Methoxybenzaldehyde against *Mycobacterium avium* subsp. *paratuberculosis* (*Map*)

**DOI:** 10.3390/foods6090072

**Published:** 2017-08-24

**Authors:** Stella W. Nowotarska, Krzysztof Nowotarski, Irene R. Grant, Christopher T. Elliott, Mendel Friedman, Chen Situ

**Affiliations:** 1 Institute for Global Food Security, School of Biological Sciences, Queen’s University Belfast, David Keir Building, Stranmillis Road, Belfast BT9 5AG, UK; snowotar@gmail.com (S.W.N.); knowotar@hotmail.com (K.N.); i.grant@qub.ac.uk (I.R.G.); Chris.Elliott@qub.ac.uk (C.T.E.); 2 Western Regional Research Center, Agricultural Research Service, U.S. Department of Agriculture, Albany, CA 94710, USA

**Keywords:** essential oils, oil compounds, benzaldehydes, *Mycobacterium avium* subsp. *paratuberculosis*, Johne’s disease, Crohn’s disease, type 1 diabetes mechanism, cell membrane, AP release

## Abstract

The antimicrobial modes of action of six naturally occurring compounds, cinnamon oil, cinnamaldehyde, oregano oil, carvacrol, 2,5-dihydroxybenzaldehyde, and 2-hydroxy-5-methoxybenzaldehyde, previously found to inhibit the growth of *Mycobacterium avium* subsp. *paratuberculosis* (*Map*) reported to infect food animals and humans and to be present in milk, cheese, and meat, were investigated. The incubation of *Map* cultures in the presence of all six compounds caused phosphate ions to leak into the extracellular environment in a time- and concentration-dependent manner. Cinnamon oil and cinnamaldehyde decreased the intracellular adenosine triphosphate (ATP) concentration of *Map* cells, whereas oregano oil and carvacrol caused an initial decrease of intracellular ATP concentration that was restored gradually after incubation at 37 °C for 2 h. Neither 2,5-dihydroxybenzaldehyde nor 2-hydroxy-5-methoxybenzaldehyde had a significant effect on intracellular ATP concentration. None of the compounds tested were found to cause leakage of ATP to the extracellular environment. Monolayer studies involving a Langmuir trough apparatus revealed that all anti-*Map* compounds, especially the essential oil compounds, altered the molecular packing characteristics of phospholipid molecules of model membranes, causing fluidization. The results of the physicochemical model microbial membrane studies suggest that the destruction of the pathogenic bacteria might be associated with the disruption of the bacterial cell membrane.

## 1. Introduction

Many naturally-occurring compounds, such as plant extracts, secondary metabolites, and phytochemicals, have been extensively evaluated for antimicrobial activity [1,2,3,4,5,6,7,8,9]. Many of them have been shown to be active against both Gram-positive and Gram-negative bacteria, including *Bacillus cereus* [10,11], *Listeria monocytogenes* [12,13], *Pseudomonas aeruginosa*, *Salmonella typhimurium* and *Staphylococcus aureus* [14,15], *Escherichia coli* [16,17], and *Campylobacter jejuni* [18,19,20]. Some natural compounds, e.g., cinnamon oil and oregano oil, have exhibited activity against pathogenic bacteria that have developed resistance to conventional antibiotics [21,22].

*Map* is a bacterial pathogen of animal health and potential public health significance [23]. As the causative agent of Johne’s disease (paratuberculosis) in domesticated ruminants, such as cattle, sheep, goats, and rabbits [24,25,26,27,28], as well as hens [29], starlings [30], and wildlife [31], it can cause chronic diarrhea, progressive weight loss, decreased milk production, and infertility in these food animals, as well as significant economic losses to farmers [32,33,34,35].

Currently there is no drug approved for the treatment of Johne’s disease. Click [36] reported that infection with the probiotic bacterium Dietzia, alone or in combination with dexamethasone, inhibited the growth of the mycobacterium in cattle. Godden, et al. [37] and Verhegghe, et al. [38] describe the use of heat-treated colostrum for reducing *Map* in dairy cows. Vaccines in development also have the potential to protect dairy herds against *Map* [39,40,41,42].

Human epidemiological and medical studies suggest that *Map* might also contribute to the etiology of human diseases. These include Crohn’s disease of the digestive tract [43,44], type 1 diabetes [45,46,47], and multiple sclerosis [48]. The cited studies suggest the need to further define the possible conflicting role of *Map* in the cause, mechanism, and prevention of *Map*-induced adverse effects in humans [49].

Once farm animals are identified as being infected with *Map*, they are culled prematurely. However, because of the relatively long latent period of *Map* infections, generally between two and five years, by the time the first clinically-affected animal is identified a significant proportion of the herd could have been infected by the bacterium. *Map* can be readily transmitted through the fecal-oral route between animals and via contaminated water because it can persist in the farm environment for lengthy periods [50,51,52,53,54]. Transmission of *Map* to humans may be via contaminated dairy products, meat, or water [55]. *Map* has been isolated from or detected in dairy products such as cheese, raw and pasteurized milk, and infant milk formula [56,57,58,59]. The bacteria can survive extreme conditions such as low pH, high pasteurization temperatures, or low refrigeration temperatures [60].

In a previous study we investigated the effect of a range of naturally-occurring compounds on *Map* cells. Six compounds (cinnamon oil, *trans*-cinnamaldehyde, oregano oil, carvacrol, 2,5-dihydroxybenzaldehyde, and 2-hydroxy-5-methoxybenzaldehyde) inhibited the growth of *Map* [61]. Following appearance of our study, Crandall et al. [62] reported that citrus oils inhibited the growth of *Mycobacterium tuberculosis* species in vitro. These authors suggested that the observed anti-mycobacterium properties of the Valencia orange oil warrant further study designed to elucidate the specific mechanisms of action. In a related study, we explored the use of monolayers of bacterial phospholipids as artificial model membranes to study the interaction these compounds with the artificial cell membranes [63].

To further define the antimicrobial mechanisms, in the present study, the potential anti-mycobacterium mechanisms of these compounds have been investigated with *Map* bacteria. Naturally-occurring compounds, such as potato and tomato glycoalkaloids and plant essential oils and their constituents are generally known to target the cell membrane of microorganisms and animal tissues owing to their hydrophobic nature, which enables them to partition into the hydrophobic part of the phospholipid bilayer and accumulate at the cell membrane [64,65]. However, the molecular interactions between the naturally-occurring compounds and bacterial cells still require further determination [15]. It has been suggested that naturally-occurring antimicrobial compounds might have several modes of action to achieve metabolic inhibition and growth inhibition of microbes, subsequently leading to cell death [66].

Methods for studying antibacterial mechanisms include: measuring the change of cell homeostasis, e.g., using fluorescent probes to measure the change of intracellular pH, the relative change of membrane potential and ATP synthesis [10]; measuring oxygen consumption [16]; proteomic studies to investigate protein expression or repression under stressful, but non-lethal, antimicrobial treatments [67]; observing changes in cell morphology after antimicrobial treatment using transmission [68] and scanning electron microscopy [69]; and measuring cell membrane stability, e.g., whole cell autolysis which could indicate whether the antimicrobials cause the disintegration of cells [70]. In the present study, we determined extracellular phosphate ion concentration, intracellular and extracellular ATP concentrations, and monitored changes in absorbance of supernatants from broth cultures exposed to the six active naturally-occurring compounds. In addition, monolayer model membrane studies using a Langmuir trough apparatus were performed. Monolayer model membrane studies are a biophysical approach to study the interaction of antimicrobials with lipid monolayers that mimic the outer surface of biological membranes. These techniques have recently been employed for mode of action studies [71,72,73]. 

Biological membranes have an extremely complex chemical composition including different types of phospholipids [74] which serve as a matrix for other essential components, e.g., proteins, including enzymes and biopolymers for cellular function [75,76,77,78]. Due to the complexity of biological membranes, different varieties of model membrane systems, e.g., liposome, planar lipid bilayer, and monolayer have been studied [79]. In this study, a monolayer model membrane was used as a single component system to analyze the influence of antimicrobial substances on lipid behavior in the membrane. We believe this to be the first reported study to determine the cellular response of *Map* to six naturally-occurring compounds previously demonstrated to inhibit growth of this bacterium. Possible modes of action of the plant antimicrobials against *Map* are discussed.

## 2. Materials and Methods 

### 2.1. Plant Materials

The naturally-occurring compounds 2,5-dihydroxybenzaldehyde, 2-hydroxy-5-methoxybenzaldehyde, carvacrol, and *trans*-cinnamaldehyde were obtained from Sigma-Aldrich (Poole, Dorset, UK); the purity levels of these compounds ranged from 98 to 99% according to the manufacturer’s specifications. Cinnamon oil (purity > 95%), containing 85% *trans*-cinnamaldehyde, was obtained from Yerba Buena Co. (Berkeley, CA, USA). Oregano oil (purity > 95%), containing 85% carvacrol, was obtained from Lhasa Karnak Herb Co. (Berkeley, CA, USA). The purity levels of 2,4,6-trihydroxybenzaldehyde, geraniol, and vanillic acid (Sigma-Aldrich, Dorset, UK); ranged from 97% to 98% according to the manufacturer’s data. Stock solutions, 50 mg/mL for solid compounds and 50 µL/mL for oil compounds, were prepared by suspension in absolute ethanol. As a high percentage of ethanol is bactericidal, the maximum concentration of ethanol that a strain could tolerate (showing no observable inhibition of growth) was determined in a previous study of the bactericidal assay [61]. The final concentration of ethanol present in all studies using *Map* culture was standardized at 0.4% (*v*/*v*).

### 2.2. Bacterial Strains and Growth Conditions

*Mycobacterium avium* subsp. *paratuberculosis* NCTC 8578 (a bovine isolate) was originally obtained from the National Collection of Type Cultures, Colindale, London. The strain was maintained in Cryobank vials (Mast Group Ltd., Merseyside, UK) at −70 °C. When a broth culture was required, two cryobeads were inoculated into 10 mL of Middlebrook 7H9 broth (Difco Laboratories, Detroit, MI, USA), supplemented with 0.05% (*w*/*v*) Tween 80 (Sigma-Aldrich, Dorset, UK), 10% (*v*/*v*) oleic albumin dextrose catalase (OADC) supplement (Difco), and 2 µg/mL of mycobactine J (Synbiotics Europe SAS, Lyon, France), pH 6.6 ± 0.2, and incubated at 37 °C with shaking at 100 rpm for 28 to 35 days until the optical density at 600 nm (OD_600_) was between 0.7 and 1.0. Fifty milliliters of early stationary phase *Map* culture (OD_600_ = 0.7–1.0, ~5 × 10^7^ cfu/mL) was centrifuged at 2500× *g* for 30 min to pellet the cells. Cell pellets were washed three times in 50 mL of sterile reverse osmosis water (Milli Q water system; Millipore, Molsheim, France) at room temperature. *Map* cells were resuspended differently depending on the analysis to be performed, as indicated below.

### 2.3. Determination of Extracellular Phosphate Concentration

The phosphate assay was carried out as described by Lambert, et al. [15] with minor modifications. Briefly, the washed *Map* cell pellets were resuspended to the original volume in sterile HPLC water (Sigma-Aldrich, Dorset, UK). Aliquots (2 mL) of the cell suspension were transferred into sterile Eppendorf tubes to which the test compounds were added. Four of the anti-*Map* compounds (cinnamaldehyde, carvacrol, 2,5-dihydroxybenzaldehyde, and 2-hydroxy-5-methoxynenzaldehyde) and a non-active compound vanillic acid, were tested at three-fold dilutions ranging from eight to 1000 µg/mL. Ethanol (0.4%, *v*/*v*) was used as negative control. The samples were incubated at 37 °C for 24 h with aliquots taken at 0, 1, 2, 4, 8, and 24 h time points. Aliquots were transferred into Eppendorf tubes and centrifuged at 16,873× *g* for 15 min. Supernatants were carefully transferred into fresh Eppendorf tubes. All samples were stored at −20 °C until used. For the phosphate test, aliquots of each sample (50 µL) were pipetted into a flat-bottom 96-well microtitre plate (Sarstedt Ltd., Leicester, UK) and Biomol Green™ phosphate assay reagent (100 µL) (Enzo Life Sciences UK Ltd., Exeter, UK) was dispensed into each well. The plate was sealed and incubated at room temperature for 20 min to allow for color development. The OD_620_ was then measured using a microplate reader (Tecan UK Ltd., Reading, UK). Phosphate standard solution (Enzo Life Sciences UK Ltd., Exeter, UK) was prepared to establish a calibration curve range from 0 to 40 µM to allow the phosphate concentration to be determined. The experiment was repeated twice on different dates.

### 2.4. Determination of Intra- and Extra-Cellular ATP Concentration

The intracellular and extracellular concentrations of ATP were investigated using the procedures described by Gill and Holley [12] with minor modifications. Briefly, the washed *Map* pellet was resuspended in 7H9 broth base (45 mL) without OADC or mycobactine J and incubated at 37 °C to starve (deplete the intracellular ATP) for six weeks. Aliquots of the cell suspension (3.6 mL) were transferred into small bottles, and supplemented with 0.5% d-glucose. Duplicate bottles were supplemented with natural compound stock (17.76 µL) to achieve the previously-determined minimum inhibitory concentration (MIC) of each compound (MIC: cinnamaldehyde, 24 mg/L; cinnamon oil, 24 mg/L, carvacrol, 74 mg/L, oregano oil, 74 mg/L; 2,5-dihydroxybenzaldehyde, 74 mg/L; and 2-hydroxy-5-methoxybenzaldehyde, 74 mg/L). Two other bottles were supplemented with the non-active compound 2,4,6-trihydroxybenzaldehyde (17.76 µL) stock to achieve a final concentration of 74 mg/L. Another two bottles were supplemented with absolute ethanol (17.76 µL) to a final concentration 0.4% *v*/*v* to serve as negative controls. At 0, 1, 2, 4, 8, and 24 h, samples were taken and subjected to the following procedure. A sample (500 µL) from each bottle was transferred to an Eppendorf tube; the cells were pelleted by centrifugation at 2500× *g* for 15 min, and the supernatant (250 µL) was carefully transferred to an Eppendorf tube containing 200 mM Tris (250 µL) + 4 mM EDTA + 0.05% dodecyl trimethyl ammonium bromide (DTAB) for stabilization and determination of extracellular ATP concentration. The remaining half of the supernatant was carefully discarded. The cell pellet was then resuspended in an ‘extractant’ (500 µL; 100 mM Tris + 2 mM EDTA + 0.025% DTAB). To facilitate ATP extraction, the cell suspensions mixtures were heated in a boiling bath for 20 min. After heat treatment, samples were placed in an ice bath to facilitate rapid cooling. Triplicate aliquots (50 µL) of each sample were pipetted into a white 96-well plate (Greiner Bio-One Ltd., Gloucestershire, UK). ATP standard (Sigma-Aldrich, Dorset, UK) at a concentration of 1, 2, 4, 8, 16, 32, and 64 nM prepared in the extractant was used to generate a calibration curve. The ATP assay mix reagent (50 μL) (Sigma-Aldrich, Dorset, UK) was dispensed into each sample and standard well. The bioluminescence was then read immediately using a microplate reader (Mithras LB 940, Berthold Technologies UK Ltd., Herts, UK). This experiment was repeated twice.

### 2.5. Absorbance Scans of Culture Supernatant after Antimicrobial Treatment

The washed *Map* cell pellets were resuspended in the original volume of sterile reverse osmosis water. Aliquots (4 mL) were transferred to small sterile bottles and supplemented with test compounds. Three of the anti-*Map* compounds (carvacrol, 2,5-dihydroxybenzaldehyde and 2-hydroxy-5-methoxynenzaldehyde) and the non-active vanillic acid were tested at 74 µg/mL, and cinnamaldehyde was tested at 24 mg/L. Since all of the natural compounds were suspended in absolute ethanol as a stock solution prior to addition to the *Map* cultures, ethanol (0.4% (*v*/*v*) was used as a negative control. The bottles were incubated at 37 °C for 48 h. At 0, 1, 2, 4, 8, 24, and 48 h, sample aliquots were pipetted into Eppendorf tubes and centrifuged at 16,873× *g* for 15 min. Supernatants were carefully transferred into fresh Eppendorf tubes. For the scan experiment, samples (200 µL) were pipetted into separate wells of a flat-bottom 96-well UV plate (Costar, Corning Ltd., Sunderland, UK). The samples were scanned from 230 nm to 370 nm with a wavelength step size of 5 nm, using a microplate reader (Tecan UK Ltd., Reading, UK). Since most of the natural compounds absorb UV light and might show more than one peak when scanned for a series of wavelengths, a corresponding blank (composed of water and natural compound only, without culture) was prepared for each natural compound and processed under the same conditions as the *Map*-containing sample to allow a comparison of supernatant samples. This experiment was repeated twice.

### 2.6. Monolayer Studies

A Langmuir trough (Precision Plus) (µTrough XL; Kibron, Helsinki, Finland) equipped with a computer-controlled microbalance (Kibron) and MicroSpot (Kibron) was used to measure surface pressure-area (π-A), using the control software (FilmWare 3.61; Kibron). The total surface area of the trough was 227.15 cm^2^, and the volume of the subphase was approximately 0.1 L. Phospholipids 1,2-di-(9Z-octadecenoyl)-*sn*-glycero-3-phosphoethanolamine (DOPE) and 1,2-di-(9Z-octadecenoyl)-*sn*-glycero-3-phospho-(1′-*rac*-glycerol) sodium salt (DOPG) were purchased from Avanti Polar Lipids (Alabaster, Alabama, USA). Stock solutions of the phospholipids (5 mg/mL) were prepared in chloroform (Sigma-Aldrich, Dorset, UK) and stored at −20 °C. The naturally-occurring antimicrobial compounds were prepared as stock solutions in reverse osmosis water for powder compounds (100 mg/mL) and chloroform for oil compounds (500 mg/mL). Reverse osmosis water was freshly obtained and used in all experiments. The stocks of powder compounds were freshly prepared before each experiment and used on the same day. The stocks of oil compounds were stored at −20 °C until required. The compounds were tested at concentrations closed to the MIC determined in previous antibacterial assays [61] was 74 mg/L for powder compounds. For the essential oil compounds, the above stock solution (1 µL) was deposited onto the surface of the subphase. The concentration could not be determined as the oil compounds were surface active and no shaking or stirring was employed to facilitate mixing. The phospholipids prepared in chloroform were deposited onto the water surface (pure water with or without natural compounds in the subphase) interface using a 5-µL Hamilton micro-syringe (Supelco, Bellefonte, PA, USA). The selection of subphases was based on the results of the previous antimicrobial assays by Wong, et al. [61]. After spreading the phospholipids, the monolayer was allowed to equilibrate for 10 min (to ensure evaporation of the solvent). Film compression was initiated by moving the two barriers symmetrically, with a speed of 21.157 Å^2^/chain/min to allow for the reorientation and relaxation of the lipids during compression. Surface pressure (π) was measured with an accuracy of ±0.1 mN/m using a metal wire probe (Kibron) linked to a high precision microbalance connected to a computer. All isotherms were recorded at 23 °C. The subphase temperature was controlled thermostatically to within 0.1 °C by a circulating water system (Grant Instruments (Cambridge) Ltd., Cambridgeshire, UK).

### 2.7. Analysis of Isotherms

The interaction between lipid molecules deposited on subphase containing different antimicrobials and their molecular organization were analyzed by calculating the compression modulus C_S_^−1^, which is a reciprocal of isothermal compressibility (C_S_) as previously described [80]. The C_S_ value of the investigated films at the given surface pressure (π) was obtained from π-A data as follows: C_S_ = (−1/A_π_) (dA/dπ)_T_, where A_π_ is the area per molecule at the indicated surface pressure π. The C_S_ values were calculated using Origin^®^ 8 program (OriginLab Corporation, Northampton, MA, USA). Accordingly, the higher the value of the compressibility modulus C_S_^−1^; the lower the interfacial fluidity will be [81].

### 2.8. Data Analysis

Graphs were plotted using the mean of the replicates of each experiment. Ordinary ANOVA was performed on data, when appropriate, by using GraphPad InStat (GraphPad Software, Inc., La Jolla, CA, USA).

## 3. Results

### 3.1. Extracellular Phosphate Assay

**Figure 1:**
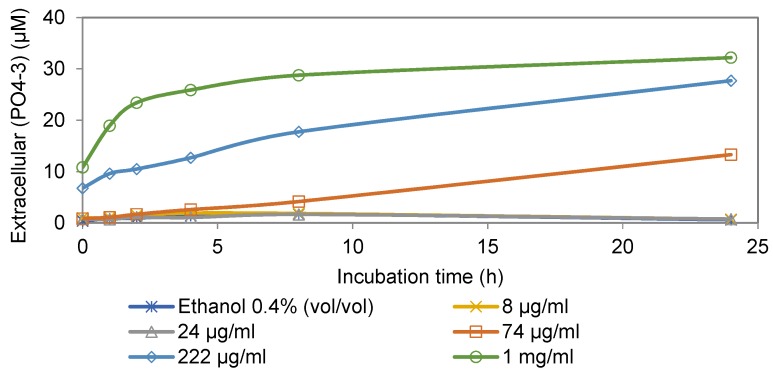
Extracellular levels of phosphate ion in aliquots of *Map* cultures incubated with various concentrations of cinnamaldehyde (MIC 24 mg/L) and non-inhibitory concentration of ethanol (0.4%, *v*/*v*) as the negative control.

**Figure 2:**
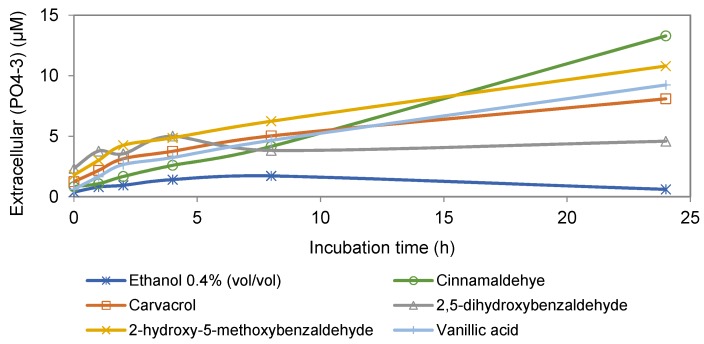
Extracellular concentration of phosphate ion in cultures of *Map* incubated with different active compounds, non-inhibitory compound (vanillic acid) at 74 mg/L, and non-inhibitory concentration of ethanol (0.4%, *v*/*v*).

The effect of cinnamaldehyde on phosphate leakage of *Map* cells is shown in Figure 1. The results showed an increase in extracellular phosphate level in the presence of cinnamaldehyde that was concentration- and time- dependent, suggesting increased membrane permeability. Figure 2 shows the effects of four anti-*Map* compounds and two negative controls, non-active compound vanillic acid and ethanol, on phosphate leakage of the *Map* culture. Similar to cinnamaldehyde, the results indicated that membrane permeability increased in the presence of all naturally-occurring compounds, including the non-active compound, and the observed increase was time-dependent. 

### 3.2. Intracellular and Extracellular ATP Assays

Cinnamon oil and its active ingredient cinnamaldehyde significantly decreased intracellular ATP (*p* < 0.05). Oregano oil and its active constituent carvacrol resulted in an initial rapid decrease of intracellular ATP concentration, before it rose again (data not shown). The final intracellular ATP concentrations of *Map* cells exposed to these two compounds were still low (*p* < 0.05) compared to the two negative controls. No significant change in intracellular ATP was observed with the other tested compounds.

Low levels of ATP were found in the extracellular environment in all the samples tested including anti-*Map* compounds and negative controls (data not shown). The extracellular ATP concentration was very low (0.5 nM) and remained unchanged throughout the incubation period. 

### 3.3. Absorbance Scans of Culture Supernatant after Antimicrobial Treatment

**Figure 3:**
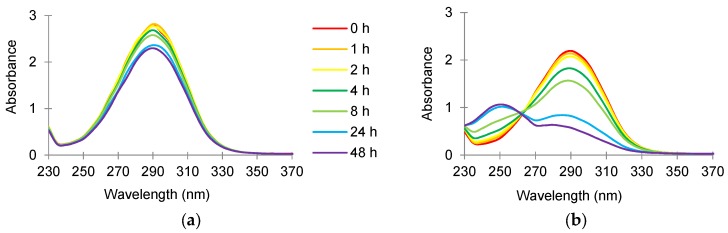
Absorbance scan (230 to 370 nm) of 24 mg/L cinnamaldehyde prepared in sterile reverse osmosis water and incubated in (**a**) absence of and (**b**) presence of *Map* NCTC 8578 at 37 °C for 48 h.

The absorbance scan (from 230 to 370 nm) of cinnamaldehyde (24 µg/mL) in sterile reverse osmosis water incubated at 37 °C is shown in Figure 3a. A peak was observed and the highest absorbance was measured at 290 nm (A_290_). The A_290_ reading represents the abundance of cinnamaldehyde in the sample, which decreased from about 2.75 to 2.25 during the two-day incubation period. When a similar scan was performed on the supernatant of the culture incubated with 24 mg/L cinnamaldehyde (the MIC that inhibits the growth of *Map* in broth medium), the A_290_ reading also decreased over the two-day incubation period (Figure 3b). A new peak, which had the highest absorbance at about 250 nm, appeared after one day of incubation. 

**Figure 4:**
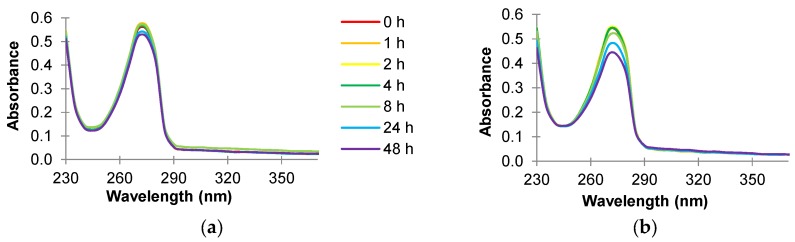
Absorbance scan (230 to 370 nm) of 74 mg/L carvacrol prepared in sterile reverse osmosis water and incubated in (**a**) absence of and (**b**) presence of *Map* NCTC 8578 at 37 °C for 48 h.

An absorbance scan (230 to 370 nm) of carvacrol (74 mg/L) in sterile reverse osmosis water or the supernatant of *Map* cultures incubated at 37 °C is illustrated in Figure 4a,b. Maximum absorbance was observed at about 270 nm (A_270_) (Figure 4a). When a similar scan was performed on the supernatant of the *Map* culture incubated with 74 mg/L carvacrol (the MIC that inhibits the growth of *Map* in broth medium) (Figure 4b), the A_270_ reading decreased as the time of incubation increased.

### 3.4. Monolayer Studies

**Figure 5:**
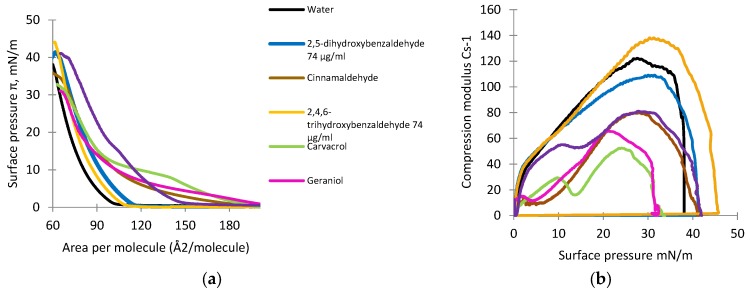
The surface pressure-area (*π-A*) (**a**) and compression modulus (C_S_^−1^) values versus surface pressure (*π*); and (**b**) isotherms recorded for monolayers formed by DOPE on the subphases containing pure water with and without naturally-occurring antimicrobial compounds.

The compression isotherms and compression modulus for the DOPE monolayer deposited on subphases containing natural antimicrobial compounds are shown in Figure 5a,b. In subphase containing pure water only, the lift-off value was approximately 105 Å^2^ per molecule and increased gradually into the liquid-condensed (LC) phase up to around 62 Å^2^ per molecule, at which point the monolayer collapsed (collapse pressure was about 38 mN/m) (Figure 5a). Analysis of the compression modulus for the DOPE monolayer in the presence of different naturally occurring antimicrobial compounds is shown in Figure 5b. 

**Figure 6:**
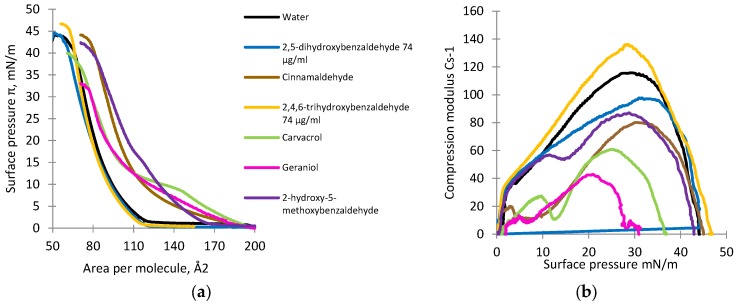
The surface pressure-area (*π-A*) (**a**) and compression modulus (C_S_^−1^) values versus surface pressure (*π*); and (**b**) isotherms recorded for the monolayers formed by DOPG on the subphases containing pure water with and without naturally occurring antimicrobial compounds.

The compression isotherms and compression modulus for the DOPG monolayer deposited on subphases containing selected naturally-occurring antimicrobial compounds are shown in Figure 6a,b. In the subphase containing pure water only, the lift-off value was approximately 120 Å^2^ per molecule and increased gradually into the liquid-condensed (LC) phase up to around 68 Å^2^ per molecule, at which point the monolayer collapsed (collapse pressure was about 42 mN/m) (Figure 6a). 

Analysis of the compression modulus for the DOPG monolayer in the presence of different antimicrobial compounds is shown in Figure 6b. 

## 4. Discussion

### Mechanisms of Antimicrobial Effects against Map

The measurement of intracellular constituents in the extracellular environment, e.g., soluble proteins [69], nucleic acids [82], ATP [83], and phosphate and potassium ions [15] are good indicators of membrane leakage and can reflect the severity of membrane damage. These cellular constituents are important for cellular functions. Presumably, leakage of these constituents might cause further disturbances in cellular metabolism and viability.

As shown in Figure 1 and Figure 2, the degrees of phosphate leakage varied between different compounds in the present study. Cinnamaldehyde caused the highest level of phosphate leakage (Figure 2) in a dose- and time-dependent manner (Figure 1). By contrast, 2,5-dihydroxybenzaldehyde induced the lowest level of leakage. Lambert, et al. [15] reported that oregano essential oil caused leakage of phosphate ion in both the Gram-positive bacterium *S. aureus* and the Gram-negative bacterium *P. aeruginosa*. However, because supplementary controls were not included, it is unclear whether the leakage observed from the two organisms was caused only by the oregano essential oil.

The effects of six anti-*Map* compounds at their MIC values on the intracellular and extracellular ATP concentration of *Map* cells that had been starved for six weeks were also investigated. Significant effects (*p* < 0.05) of cinnamon oil and cinnamaldehyde were observed. The transient effect (an initial rapid decrease) of oregano oil and its active ingredient carvacrol (data not shown), suggests that oregano oil and carvacrol might exert their antimicrobial effect by means of initially stressing *Map*. The data show that *Map* was able to partially overcome such a challenge and to decrease the effect to some degree. Thus ATP synthesis might have resumed or ATP hydrolysis might have slowed down as a result of a self-defense mechanism. In the present study, we found that carvacrol did not deplete the intracellular ATP pool of *Map*. This finding contrasts with observation that carvacrol rapidly depleted the ATP pool in the Gram-positive foodborne pathogen *Bacillus cereus* [10].

A possible explanation for the different effects might be due to the differences in the cell membrane compositions. The *Map* membrane is characterized by a thicker, waxy, and hydrophobic cell wall, which is very rich in mycolic acid, making it more resistant to chemical damage and environmental stress. Low levels of extracellular ATP found in our study may be due to a weak ATP efflux as a result of *Map* being stressed when exposed to the antimicrobial compounds. This also implies that the compounds did not increase the membrane permeability for ATP.

The absorbance scan of cinnamaldehyde (24 μg/mL) in water or *Map* culture showed a decrease of the peak at 290 nm over the two-day incubation period (Figure 3a,b). When comparing the culture supernatant at time 0 min with the corresponding blank, the A_290_ reading is approximately 0.5 absorbance units lower in the supernatant. The explanation for this finding might be due to the adsorption of cinnamaldehyde to the cell surface or uptake of cinnamaldehyde into the cells. Furthermore, a new peak, which had the highest absorbance at about 250 nm, appeared after one day. The new peak could either be a product transformed by *Map* by cinnamaldehyde during the incubation, or an intracellular constituent that had leaked from the cells. Since the peak showed the highest absorbance within the UV region (250 nm), it is possibly an indication of nucleic acid or leakage of other constituents from the cells. The appearance of such a new peak was unique to cinnamaldehyde; culture supernatants from cells exposed to all of the other compounds did not show additional peaks. Attempts were made to identify this peak, using QToF LC/MS and NMR spectroscopy, but without success.

An absorbance scan (230 to 370 nm) of carvacrol in sterile reverse osmosis water (Figure 4a) or supernatant cultures (Figure 4b) shows that the highest absorbance was observed at 270 nm (A_270_). The A_270_ value decreased from about 0.58 to 0.53 during the two-day incubation period (Figure 4a), similar to the absorbance scan of cinnamaldehyde in water. However, the decrease in A_270_ was much smaller than that with cinnamaldehyde. When a similar scan was performed on the supernatant of the *Map* culture incubated with carvacrol (74 mg/L; the MIC that inhibits the growth in broth medium) (Figure 4b), the A_270_ reading decreased as the time of incubation increased, i.e., from about 0.55 to 0.44. The decreased quantity of carvacrol suggested the uptake of carvacrol by the *Map* organism. However, in contrast to the cinnamaldehyde experiment, the decrease in absorbance reading in culture supernatant treated with carvacrol was much smaller and no additional peaks were observed.

Lipid monolayers were formed at the air-liquid interface to mimic the outer surface of the bacterial membrane by using DOPE and DOPG. PE and PG phospholipids were selected for this study because they are the predominant zwitterionic and anionic phospholipids found in the membranes of both Gram-positive and Gram-negative bacteria [75,78]. The changes in compressibility modulus in both the DOPE and DOPG monolayers reflect the physical state of the lipid monolayer compressed on the surface of different subphases. The factor that determines compressibility of the isotherm is its slope. This can be calculated as a derivative of area per molecule versus surface pressure, which reflects the rate of change of the area per molecule against surface pressure. The higher the value of the compressibility modulus, the higher the rigidity of the model membrane and, vice versa, a low value of compressibility modulus indicates high fluidity of the model membrane [84].

Isotherms of DOPE deposited on the subphase with naturally-occurring antimicrobial compounds showed the lift-off value increased and varied from approximately 110 to 200 Å^2^ per molecule (Figure 5a). The increase in the lift-off value indicated these antimicrobial compounds could have been incorporated into the lipid monolayers in the gas state resulting in the observed reduced packing effectiveness of DOPE molecules. In the presence of three oil compounds, cinnamaldehyde, carvacrol, and geraniol, the slopes of the isotherms were the flattest. For the DOPE monolayer compressed on the subphase containing pure water only, the maximal value of C_S_^−1^ was observed at approximately 122 mN/m, whereas the value for 2,4,6-trihydroxybenzaldehyde was approximately 138 mN/m, suggesting a rigidifying effect of this compound on the DOPE monolayer (Figure 5b). For the other compounds, the maximal values of the compression modulus varied from 50 to 110 mN/m. Such a decrease in the maximal value of the compression modulus indicated a fluidizing effect possibly in the DOPE monolayer. Carvacrol showed the highest fluidizing effect, whereas 2,5-dihydroxybenzaldehyde exhibited the lowest effect.

Isotherms of DOPG deposited on the subphase with natural antimicrobial compounds showed the lift-off value varied from approximately 117 to 195 Å^2^ per molecule (Figure 6a). In the presence of two of the oil compounds, carvacrol and geraniol, the slopes of the isotherms were the flattest. 2,4,6-Trihydroxybenzaldehyde and 2,5-dihydroxybenzaldehyde caused a non-significant decrease in the lift-off value (*p* > 0.05). For the DOPG monolayer compressed on a subphase containing pure water only, the maximal value of C_S_^−1^ was observed at approximately 118 mN/m, whereas the value for 2,4,6-trihydroxybenzaldehyde was approximately 137 mN/m, indicating a rigidifying effect (Figure 6b). For the other compounds, the maximal values of the compression modulus varied from 42 to 117 mN/m. Geraniol showed the highest fluidizing effect, whereas 2,5-dihydroxybenzaldehyde showed the lowest effect.

It has been reported that antimicrobial compounds interact more easily with the gas or liquid phase of the monolayer than with the liquid-condensed phase [85]. The shape and size of lipid rafts/aggregates seem to be determined by competition between the line tension at the raft boundary and the electrostatic repulsion between molecular polar heads [86]. The present biophysical experiment indicated that antimicrobial compounds interacted differently with the lipid rafts in both lipids, depending on the electrical charge at the polar head as indicated by the higher lift-off values. The difference might be due to the electrostatic interaction between charged head groups and antimicrobials inserted between hydrophobic tails.

In the present study, only 2,4,6-trihydroxybenzaldehyde caused a slight rigidifying effect on both DOPE and DOPG. All other compounds exhibited either a small or large fluidizing effect. The essential oil compounds carvacrol and geraniol showed a greater fluidizing effect in all the monolayers tested than did the solid compound 2,5-dihydroxybenzaldehyde. Sikkema, et al. [87] suggested that the toxicity of a compound is highly related to its ability to disturb the hydrophobic interactions between the lipids and proteins in the cell membrane. The toxicity is, thus, highly related to the decline in membrane integrity. The present results on the relationship between antimicrobial and membrane effects are consistent with this suggestion.

It has been suggested that hydrophobic oil compounds interact with the bacterial cell membrane, changing the membrane structure, stability, and permeability of certain intracellular constituents. Results of this study clearly indicated that natural antimicrobial compounds could modify the lipid monolayer structure, leading to the leakage of phosphate and other essential cell components and a change in the membrane potential and the cell environments, ultimately causing the death of cells. A related study showed that exposure of *Bacillus subtilis* ATCC 6633 cells to *Trachyspermum ammi* essential oil caused an instant loss of cytoplasm membrane integrity causing it to become increasingly permeable to protons and ions that might be responsible for the antibacterial activity [88]. Studies with green tea catechins have showed that hydrogen bonding of phenolic hydroxyl groups of catechins to cell membranes might be correlated with antimicrobial and anti-cancer-cell effects of catechins [89,90,91].

## 5. Conclusions

To conclude, the cellular responses of the potentially zoonotic pathogen *Map* in the presence of naturally-occurring compounds were studied. The natural compounds tested caused the leakage of phosphate ions from *Map* cells in relation to the time of exposure to and concentration of the test compounds; however, the extent of phosphate leakage was not consistent with the relative antimicrobial activities of the compounds. Exposure of *Map* cells to cinnamon oil or its constituent cinnamaldehyde caused a decline in intracellular ATP concentration; however, none of the naturally-occurring compounds caused a leakage of ATP to the extracellular environment. Exposure to cinnamaldehyde may have resulted in a leakage of intracellular constituents, possibly protein or nucleic acids, or the transformation to a new product by *Map*. The monolayer model membrane studies showed that natural antimicrobial compounds could modify the lipid monolayer structure by incorporation into the monolayer, formation of aggregates/rafts of antimicrobials and lipids, and reduction in the packing effectiveness of the lipid molecules, resulting in an increase in membrane fluidity. The monolayer studies, therefore, confirmed that the antimicrobial compounds, especially oil compounds, are targeting the cell membrane. The described studies offer insights into the interaction between antimicrobials and membrane lipids and identifying characteristics or actions of the most potent compounds.
